# Preoperative intravenous glucocorticoids can decrease acute pain and postoperative nausea and vomiting after total hip arthroplasty: A PRISMA-compliant meta-analysis

**DOI:** 10.1097/MD.0000000000008804

**Published:** 2017-11-27

**Authors:** Qing Yang, Zhi Zhang, Wenqi Xin, Aixiang Li

**Affiliations:** Department of Anesthesiology, Huaihe Hospital, Henan University, Kaifeng, China.

**Keywords:** glucocorticoids, meta-analysis, pain, total hip arthroplasty

## Abstract

**Background:**

A systematic review and meta-analysis of published randomized controlled trials (RCTs) were performed to assess the efficacy and safety of preoperative intravenous glucocorticoids versus controls for the prevention of postoperative acute pain and postoperative nausea and vomiting (PONV) after primary total hip arthroplasty (THA).

**Methods:**

A computer literature search of electronic databases, including PubMed, Embase, the Cochrane Central Register of Controlled Trials (CENTRAL), Web of Science, China National Knowledge Infrastructure (CNKI), and China Wanfang database, was conducted to identify the relevant RCTs comparing preoperative intravenous glucocorticoids versus placebos for reducing acute pain and PONV in THA patients. The primary outcomes included the use of the visual analog scale (VAS) with rest or mobilization at 6, 24, 48, and 72 hours and the occurrence of PONV. The secondary outcome was total morphine consumption. We calculated the risk ratio (RR) with a 95% confidence interval (95% CI) for dichotomous outcomes, and the weighted mean difference (WMD) with a 95% CI for continuous outcomes.

**Results:**

Pooled data from 7 RCTs (411 THAs) favored preoperative intravenous glucocorticoids against acute pain intensity at 4, 24, and 48 hours (*P* < .05). There was no significant difference between the VAS with rest or mobilization at 72 hours (*P* > .05). Subsequently, preoperative intravenous glucocorticoids provided a total morphine-sparing effect of 9.36 mg (WMD = −9.36, 95% CI = −12.33 to −6.38, *P* = .000). In addition, preoperative intravenous glucocorticoids were associated with a significant reduction of the occurrence of PONV (RR = 0.41, 95% CI = 0.30–0.57, *P* = .000).

**Conclusion:**

Intravenous glucocorticoids can decrease early pain intensity and PONV after THA. However, the low number of studies and variation in dosing regimens limits the evidence for its use. Thus, more high-quality RCTs are still needed to identify the optimal drug and the safety of intravenous glucocorticoids.

## 1 Introduction

Total hip arthroplasty (THA) is an excellent surgery for patients with end-stage hip osteoarthritis or arthrodysplasia.^[1,2]^ The number of THAs has been estimated to be 572,000 in the year 2030.^[3]^ THAs are always accompanied by moderate to severe postoperative pain and postoperative nausea and vomiting (PONV), resulting in poor clinical outcome and patient dissatisfaction following THA.^[4,5]^ The incidence of PONV after THA has ranged from 20% to 83%.^[6,7]^ Patient-controlled analgesia (PCA) and systemic morphine has typically been applied to control postoperative pain following THA.^[8]^ Even so, the frequency of PONV has been estimated to be approximately 37% in those treated with systemic morphine.^[9]^ The principal anesthesia indication has been used in the prevention of postoperative acute pain and the occurrence of PONV.^[10]^

Glucocorticoids have anti-inflammatory and immune-modulating properties and even prolong the postoperative analgesic effects compared with placebo.^[11]^ Thus, glucocorticoids have been used as an adjunct in these multimodal strategies. The immediate analgesic and antiemetic benefits of glucocorticoids in THA have been controversial.^[7]^ Although some randomized controlled trials (RCTs) have suggested that intravenous glucocorticoids significantly reduced pain and PONV in patients who underwent THA, due to the relatively small number of participants, their results are inconclusive.^[12,13]^ On the basis of the current clinical studies, it was necessary to conduct a meta-analysis to compare the efficacy and safety of preoperative intravenous glucocorticoids in THA patients. The purpose of the current systematic review and meta-analysis was to confirm the efficacy and safety of preoperative intravenous glucocorticoids for acute pain and PONV after THA.

## 2 Materials and methods

### 2.1 Search strategy and study selection

Electronic databases, PubMed, EMBASE, the Cochrane Central Register of Controlled Trials (CENTRAL), Web of Science, China National Knowledge Infrastructure (CNKI), and Chinese Wanfang database, were systematically searched from inception to November 6, 2016. The search strategy in PubMed was as follows: (((((((“Glucocorticoids”[Mesh]) OR dexamethasone) OR methylprednisolone) OR prednisolone) OR hydrocortisone) OR Glucocorticoids)) AND (((((“Arthroplasty, Replacement, Hip”[Mesh]) OR THR) OR THA) OR total hip replacement) OR total hip arthroplasty). There were no restrictions on language or publication status. Relevant review studies and reference lists were also manually searched for additional relevant missing studies. When the full-length article was not available from the databases, we contacted the author by e-mail or telephone to ask for it. A meta-analysis consists of collecting relevant data from published papers and thus no ethical approval was needed.

### 2.2 Eligibility criteria and exclusion criteria

Trials could be eligible for inclusion if they met the following criteria (PICOS): Participants (P): patients who have undergone THA; Interventions (I): patients administered preoperative intravenous glucocorticoids for pain control as an intervention; Comparisons (C): patients administered placebo or nothing as a control; Outcomes (O): patients with pain scores on a 110-point visual analog scale (VAS) at 6, 24, 48, and 72 hours after operation and studies that included total morphine consumption and the total occurrence of PONV; and Study design: only RCTs.

Studies were excluded if studies had incomplete data, patients had combined another strategy to control postoperative pain, and participants with a known allergy to any type of glucocorticoids.

### 2.3 Data extraction and outcome measures

Two authors (QY and ZZ) independently extracted general characteristics from the eligible studies and recorded them in a pre-generated MicrosoftExcel (Microsoft Corporation, Redmond, WA) spreadsheet. General characteristics included the first author's name, number of patients, the ratio of male patients, anesthesia method, surgery, and approach. For the intervention, study type, outcomes, and follow-up were also recorded. If the data were presented in figures, we extracted values from the diagrams using the “GetData Graph Digitizer” software (Getadata Corporation, China) as needed.^[14]^ After finishing the process of data extraction, any disagreement was resolved by discussion or a senior reviewer was consulted.

### 2.4 Risk of bias assessment

Two reviewers (W-QX and A-XL) independently evaluated the risk of bias of included studies. The study quality included the following 7 domains: random sequence generation, allocation concealment, blinding of participants and personnel, blinding of outcome assessment, incomplete outcome data, selective outcome reporting, and other biases. Domains were rated as low, high, or unclear risk of bias according to the instruction of the Cochrane Handbook for Systematic Reviews of Interventions (version 5.3.0).^[14]^ If all domains were low, the summarized risk of bias was rated low; if one or more domains were high, the summarized risk was rated high; and if one or more domains were unclear with no high-risk domains, the summarized risk was rated unclear.

### 2.5 Statistical analysis

For VAS with rest or mobilization at 6, 24, 48, and 72 hours and total morphine consumption, the weighted mean difference (WMD) and 95% confidence interval (95% CI) were calculated. For dichotomous outcomes (the occurrence of PONV), we calculated the relative risk (RR) and 95% CI. Heterogeneity was considered to be statistically significant if the *I*^2^ value was greater than 50%. A fixed-effects model was applied if the *I*^2^ value was less than 50%. Funnel plots and Egger linear regression test was performed to test the publication bias. All statistical analyses were conducted using Stata 12.0 (Stata Corp., College Station, TX). The impact of glucocorticoid dose was assessed using dexamethasone as the reference drug using an online steroid equivalence converter (http://www.medcalc.com). The relationship between glucocorticoid dosage and the relative decrease occurrence of PONV was explored using GraphPad Prism software (Version 6.0; GraphPad Software, San Diego, CA). The correlation coefficient (*r*) was used to evaluate the relationship between glucocorticoid dosage and the relative decreasing occurrence of PONV compared with the control groups. A subgroup analysis was also performed according to the dexamethasone dose (or equivalent). An equivalent dexamethasone dose less than 15 mg was identified as low dose and ≥15 mg was identified as high dose.^[15]^ A *P* value less than .05 was considered statistically significant. Mixed meta-regression (methods of moment) was used to assess any potential interaction between dexamethasone dose (or equivalent) in the first 24 hours and the risk of PONV.

## 3 Results

### 3.1 Search results

**Figure 1:**
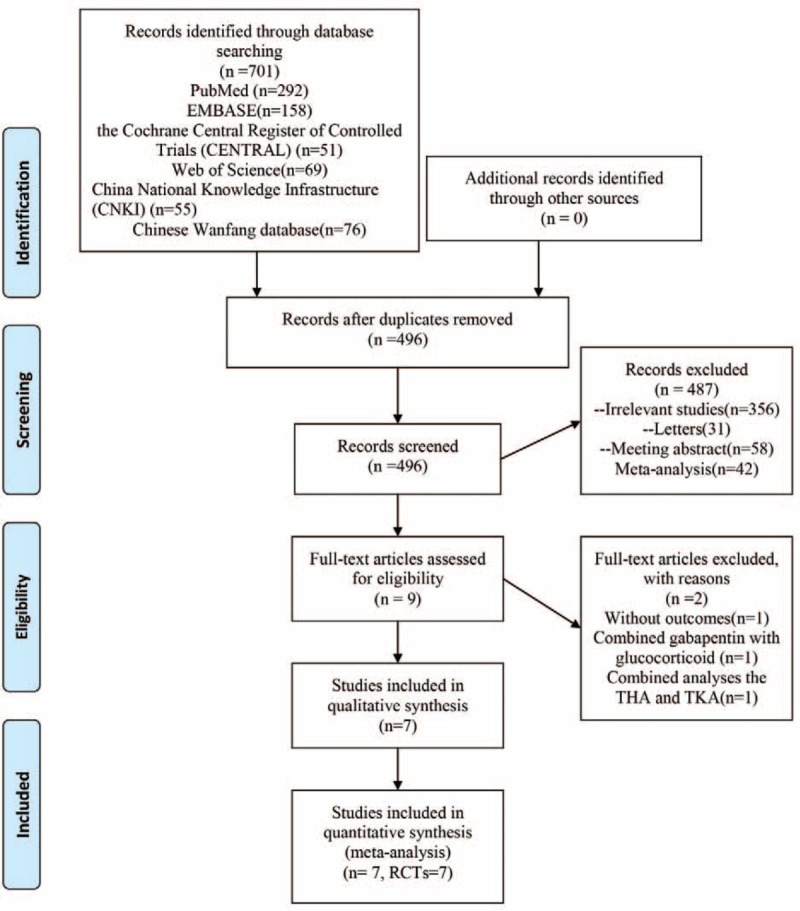
Study selection flowchart.

The literature search and selection process are shown in Fig. 1. A total of 701 relevant studies were identified initially [PubMed (n = 292), EMBASE (n = 158), the Cochrane Central Register of Controlled Trials (CENTRAL) (n = 51), Web of Science (n = 69), China National Knowledge Infrastructure (CNKI) (n = 55), and Chinese Wanfang database (n = 76)]. Then, 496 papers were reviewed after duplicates were excluded by the Endnote Software (Version X7; Thompson Reuters, CA). According to the inclusion criteria, 487 papers were excluded at the title and abstract level as the topic of these articles were irrelevant to this meta-analysis. Next, a total of 2 studies were excluded. One study^[16]^ was excluded for comparing total joint arthroplasty [total knee arthroplasty (TKA) and THA]. Another study compared combined gabapentin, ketamine, and dexamethasone versus a placebo in THA; however, the multimodal anesthesia may be different with dexamethasone alone and was thus excluded.^[17]^ Finally, we included 7^[13–23]^ RCTs (411 THAs, glucocorticoids = 240, controls = 171) for meta-analysis. The detailed baseline characteristics of the included studies are presented in Table 1. All of the articles were published between 2008 and 2016. The sample size ranged from 13 to 40 (total = 255), and the mean age ranged from 66 to 68 years. There were a total of 3 different glucocorticoids (dexamethasone,^[13–20]^ methylprednisolone,^[21]^ and hydrocortisone^[18]^) in the included studies. The follow-up time ranged from 24 hours to 1 year.

**Table 1:**
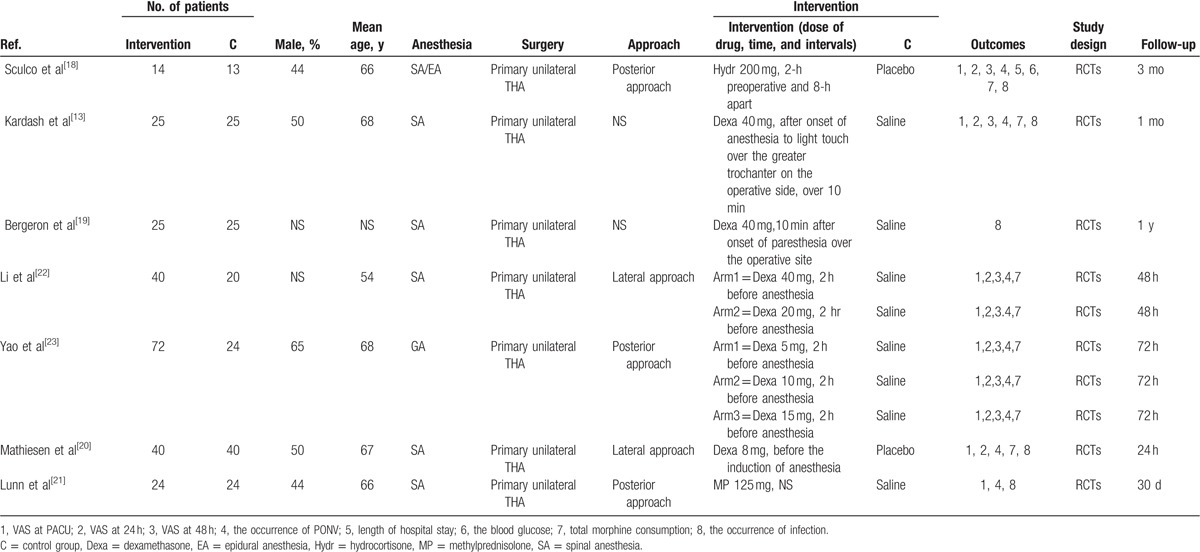
The general characteristic of the included studies.

### 3.2 Risk of bias

**Figure 2:**
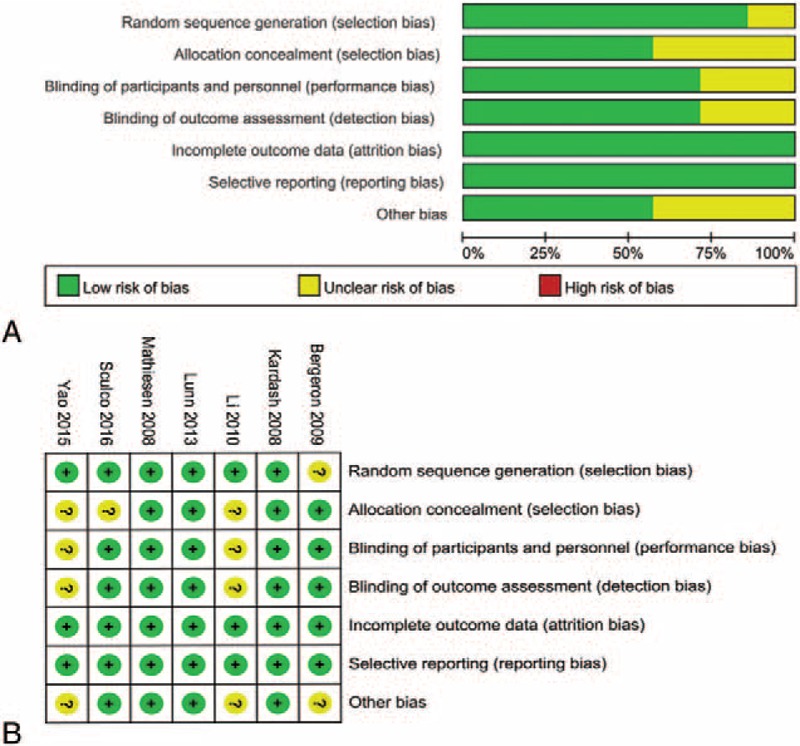
(A) The risk of bias graph; (B) The risk of bias summary; “+” represents a low risk of bias, “?” represents an unclear risk of bias; and “-” represents a high risk of bias.

**Figure 3:**
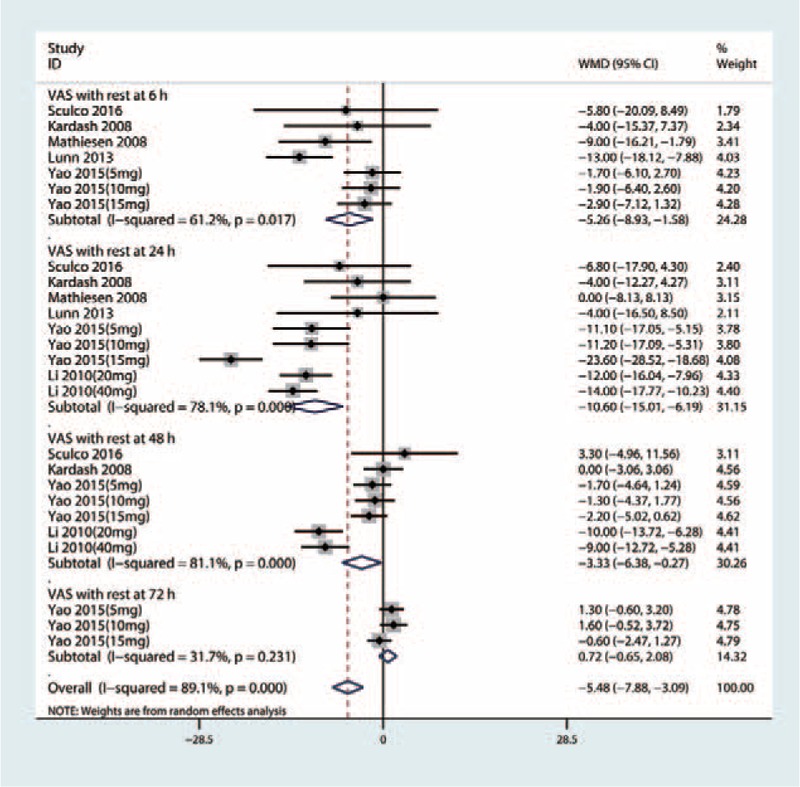
Forest plots of the included studies comparing the VAS with rest at 6, 24, 48, and 72 hours.

The details of the risk of bias assessment for all of the included studies are shown in Figs. 2 and 3. Randomized sequence generation was implemented adequately in 6 studies. However, 1 study^[19]^ had a low bias^[13–23]^ and did not report the random sequence generation and thus had an unclear risk bias. Allocation concealment was implemented adequately in 4 studies,^[13–21]^ and the rest were unclear of bias.^[18–23]^

### 3.3 Primary outcomes

#### 3.3.1 VAS with rest at 6, 24, 48, and 72 hours

Six studies,^[13–23]^ including 205 patients with THA, tested the effect of intravenous glucocorticoids on the VAS with rest at 6 hours. Compared with the placebo, intravenous glucocorticoids were associated with a significant reduction in the VAS with rest at 6 hours (WMD = −7.59, 95% CI −11.16 to −3.52, P = 0.000, Fig. 3) with large heterogeneity (*I*^2^ = 79.0%, *P* = .000).

Six studies^[13–23]^ involving 349 THAs were finally available in this meta-analysis to estimate the efficacy of preoperative intravenous glucocorticoids on the VAS with rest at 24 hours. There was a large heterogeneity (*I*^2^ = 78.1%, *P* = .000) between the included studies and thus a random-effect model was performed. Pooled results indicated that preoperative intravenous glucocorticoids were associated with a significant reduction of pain scores by 10.60 on a 110-VAS (WMD = −10.60, 95% CI −15.01 to −6.19, *P* = .000, Fig. 3).

Four studies^[13–23]^ (281 THAs) were pooled to evaluate the efficacy of preoperative intravenous glucocorticoids on the VAS with rest at 48 hours. There was a large heterogeneity between the included studies (*I*^2^ = 81.1%, *P* = .000), and thus, a random model was performed. Pooled results indicated that preoperative intravenous glucocorticoids were associated with a significant reduction on the VAS at 48 hours (WMD = −3.33, 95% CI −6.38 to −0.27, *P* = .033, Fig. 3). Three studies reported the data on the VAS with rest at 72 hours and pooled results indicated that there was no significant difference between the VAS with rest at 72 hours (WMD = 0.72, 95% CI −0.65 to 2.08, *P* = .306, Fig. 3).

#### 3.3.2 VAS with mobilization at 6, 24, 48, and 72 hours

**Figure 4:**
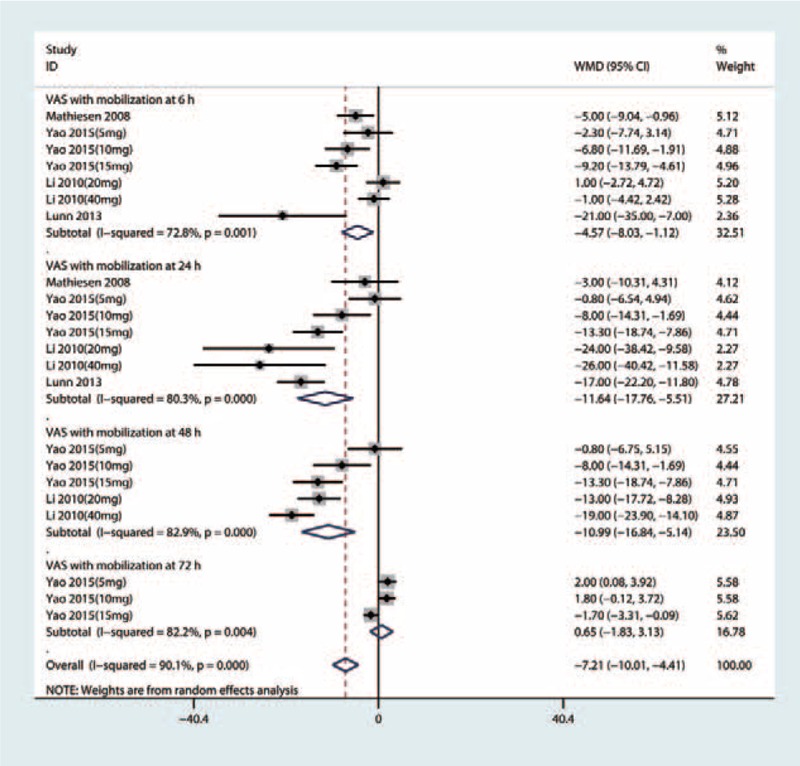
Forest plots of the included studies comparing the VAS with mobilization at 6, 24, 48, and 72 hours.

Four studies including 205 patients with THA tested the effect of intravenous glucocorticoids on the VAS with mobilization at 6 hours. Compared with the placebo, intravenous glucocorticoids were associated with a significant reduction in the VAS with mobilization at 6 hours (WMD = −4.57, 95% CI −8.03 to −1.12, *P* = .009, Fig. 4) with large heterogeneity (*I*^2^ = 72.8%, *P* = .001).

Four studies including 205 patients with THA tested the effect of intravenous glucocorticoids on the VAS with mobilization at 24 hours. Compared with the placebo, intravenous glucocorticoids were associated with a significant reduction in the VAS with mobilization at 24 hours (WMD = −11.64, 95% CI −17.76 to −5.51, *P* = .000, Fig. 4) with large heterogeneity (*I*^2^ = 80.3%, *P* = .000).

Four studies, which included 205 patients with THA, tested the effect of intravenous glucocorticoids on the VAS with mobilization at 48 hours. Compared with the placebo, intravenous glucocorticoids were associated with a significant reduction in the VAS with mobilization at 48 hours (WMD = −10.99, 95% CI −16.84 to −5.14, *P* = .000, Fig. 4) with a large heterogeneity (*I*^2^ = 82.9%, *P* = .000).

There was a large heterogeneity (*I*^2^ = 82.2%, *P* = .004) between the VAS with mobilization at 72 hours, and thus, a random-effect model was performed. There was no significant difference between the VAS with mobilization at 72 hours (WMD = 0.65, 95% CI −1.83 to 3.13, *P* = .607, Fig. 4) between the glucocorticoids group and the control group.

#### 3.3.3 The occurrence of PONV

**Figure 5:**
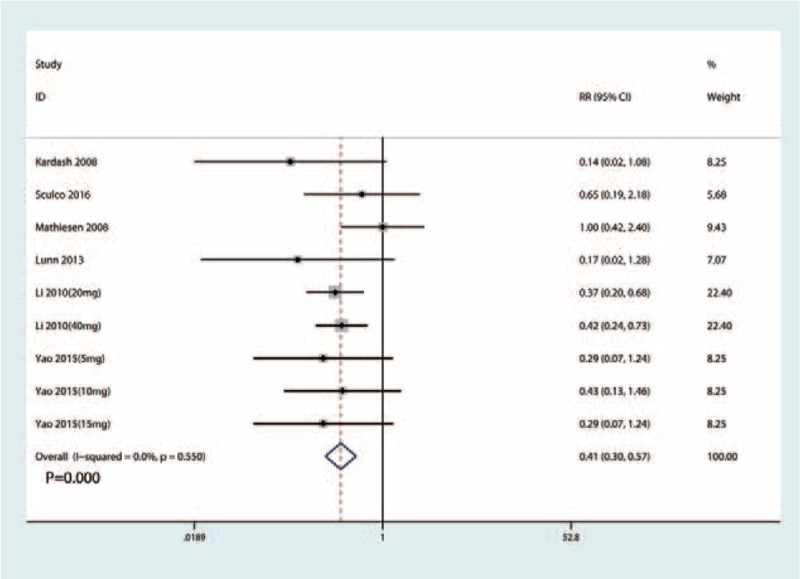
Forest plots of the included studies comparing the occurrence of PONV.

Six studies^[13–23]^ (205 participants) reported data on the occurrence of PONV. Compared with the placebo, intravenous glucocorticoids significantly decreased the occurrence of PONV (RR = 0.41, 95% CI 0.30–0.57, *P* = .000; *I*^2^ = 0.0%, Fig. 5).

**Figure 6:**
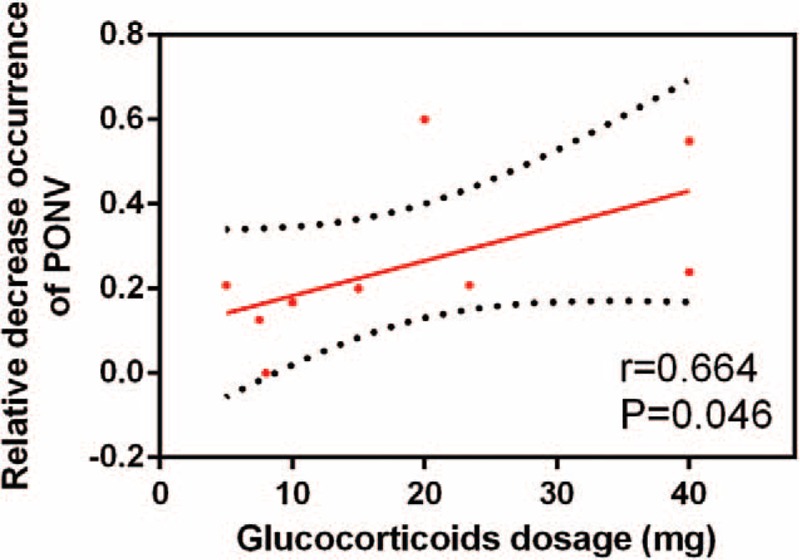
Scatter plot showing the relationship between the changing of glucocorticoid dose and the occurrence of PONV.

**Figure 7:**
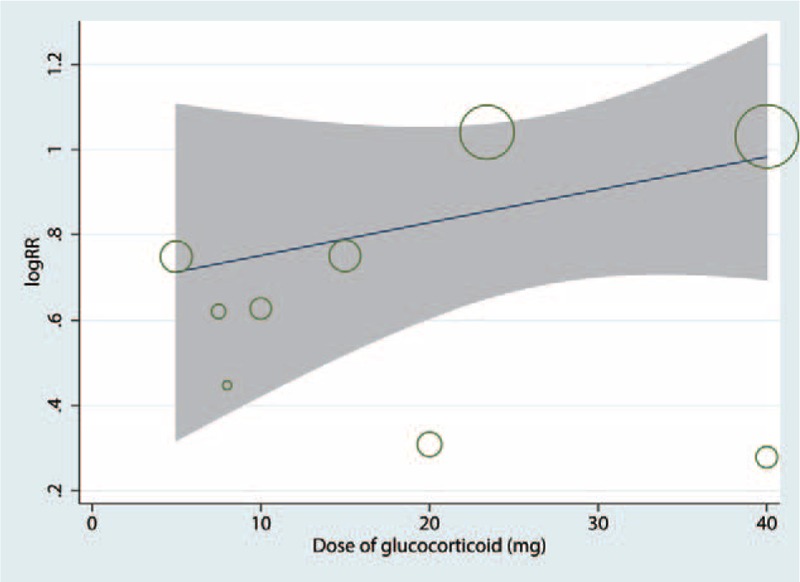
Meta-regression of the dose and the occurrence of PONV.

We plotted the glucocorticoid dose on the abscissa and the corresponding occurrence of PONV to generate a scatterplot. In addition, the linear correlation coefficient (*r*) was also calculated. A significantly positive correlation between the dosage of glucocorticoids and the occurrence of PONV was found (*r* = 0.664, *P* = .046; Fig. 6). The relative decreasing occurrence of PONV tended to increase as the glucocorticoid dose increased. Meta-regression results were in accordance with the dose–effect relationship, and the dose of glucocorticoid was an influencing factor of PONV (Fig. 7).

### 3.4 Secondary outcomes

#### 3.4.1 Total morphine consumption

**Figure 8:**
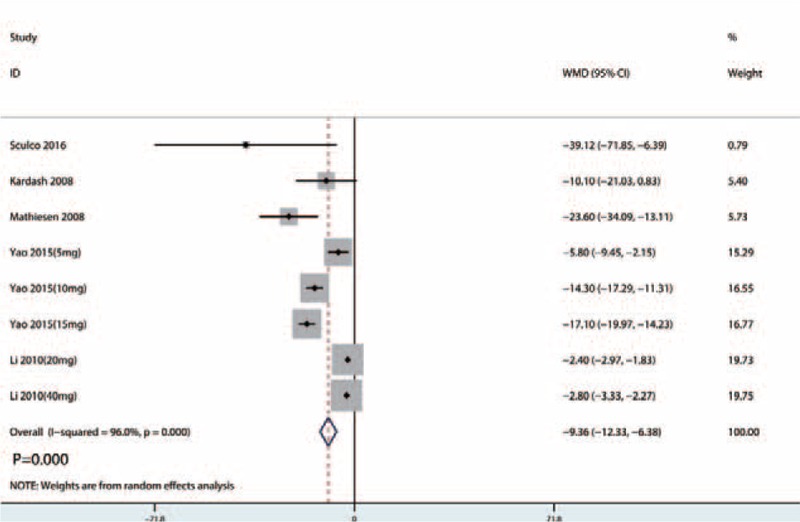
Forest plots of the included studies comparing the total morphine consumption.

A total of 5 studies^[13–23]^ (157 patients) were included in the meta-analysis of total morphine consumption. Compared with the placebo, intravenous glucocorticoids were associated with a significantly decreased total morphine consumption by 9.36 mg (WMD = −9.36, 95% CI −12.33 to −6.38, *P* = .000; *I*^2^ = 96.0%) (Fig. 8).

#### 3.4.2 Complications and functional outcome

There was a limited number of included studies that reported glucocorticoid-related complications. A summary of the complications is summarized in Table 2. Sculco et al^[18]^ reported that mean postoperative serum glucose was significantly elevated in the glucocorticoids group on the day of surgery. However, there was no significant difference between the serum glucose on postoperative days 2 and 3. Bergeron et al^[19]^ reported the Harris score of the hip, and total Harris scores were similar (*P* = .100) in the glucocorticoids group and the control group at 6 weeks and 1 year. There were no patients in either the glucocorticoids group or the control group who were subjected to the infections.

**Table 2:**
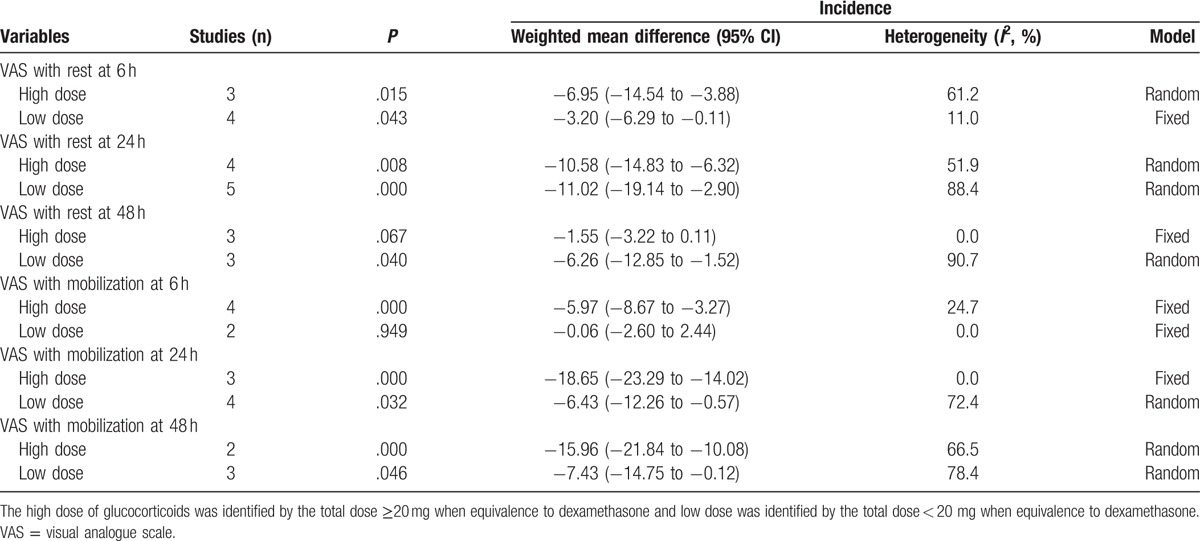
Subgroup analysis of the VAS with rest or mobilization at 6, 24, and 48 h with different dose of glucocorticoids.

#### 3.4.3 Publication bias and subgroup analysis

**Figure 9:**
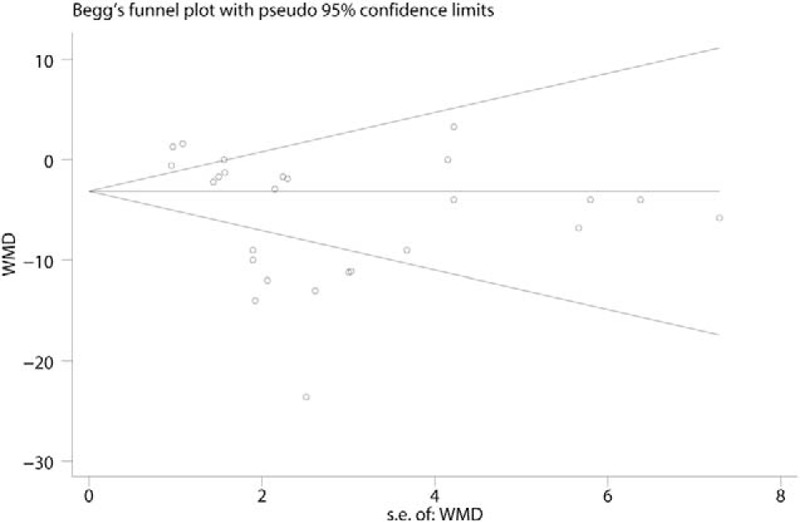
Begg test of the VAS with rest at 6, 24, 48, and 72 hours.

Begg test results for VAS with rest at 6 hours can be seen in Fig. 9. The *P* value of Begg test for VAS with rest at 6 hours was .102. Result indicated that no publication bias existed. Subgroup analysis results can be found in Table 2. A high dose of glucocorticoids was more effective in reducing VAS with rest or mobilization at 6, 24, 48, and 72 hours than a low dose of glucocorticoids.

## 4 Discussion

This is the first systematic review and meta-analysis that compares the efficacy and safety of preoperative intravenous glucocorticoids adjunct with multimodal anesthesia for patients prepared for primary THA. After a systematic search of the databases, PubMed, EMBASE, the Cochrane Central Register of Controlled Trials (CENTRAL), Web of Science, China National Knowledge Infrastructure (CNKI), and Chinese Wanfang database, a total of 7 RCTs with 411 THAs were finally included in this meta-analysis. The results indicated that intravenous glucocorticoids, compared with the placebo, were associated with a significant reduction in the VAS with rest or mobilization at 6, 24, and 48 hours; the occurrence of PONV; and total morphine consumption. There was no significant difference between VAS with rest or mobilization at 72 hours. Meanwhile, we found that the relative decrease of PONV increased as the dose of glucocorticoids increased. Moreover, there were no significant differences between the complications and hip function outcomes. The level of evidence, which was undermined by heterogeneity or the sample size limitations, was moderate or low, indicating that the degree of benefit must be studied, although the benefit is conclusive.

Intravenous glucocorticoids had a beneficial role on the VAS with rest or mobilization at 6, 24, 48 hours postoperatively. There was no significant difference between the VAS with rest or mobilization at 72 hours postoperatively. These outcomes indicated that the duration of preoperative intravenous glucocorticoids was limited in the first 48 hours. De Oliveira et al^[24]^ revealed that a preoperative single dose of intravenous glucocorticoid appeared to be effective in reducing postoperative pain without increasing the glucocorticoid-related complications. However, that study included all types of surgeries and therefore, whether preoperative intravenous glucocorticoids have a certain beneficial role on reducing pain scores and morphine-sparing effects among patients only with THA is unknown. A meta-regression and dose-relationship was also performed to identify whether the glucocorticoid dose was correlated with the reduction of pain intensity and PONV.

Intravenous glucocorticoids also had a beneficial effect in reducing the occurrence of PONV. Furthermore, the dose–effect relationships were also observed in these results. Our findings have important clinical implications because intravenous glucocorticoids are commonly given intraoperatively at the time of anesthesia induction to reduce PONV.^[25]^ A previous recent meta-analysis including 60 RCTs with 6696 subjects indicated that it favors the 4-mg to 5-mg dose regimen of systemic dexamethasone to reduce the occurrence of PONV.^[26]^ Fujii and Nakayama^[27]^ found that the rates of emesis-free effects were higher in 8 and 16 mg dexamethasone than in 4 mg dexamethasone. Awad et al^[28]^ performed an updated meta-analysis and found that intravenous dexamethasone to antiemetic drugs increases their prophylactic effect against PONV after laparoscopic cholecystectomy.

Safety concerns focus on the potential for hyperglycemia and increased infection. Blood glucose alterations were specifically mentioned in only 1 study, limiting any safety assessment on this important side effect. Nurok et al^[29]^ performed a retrospective study that included 625 patients and revealed that there was no evidence of an association between perioperative glucocorticoid administration and the odds of a high blood glucose level. Wound healing and infection were specifically mentioned in 4 studies, and none of the patients presented with infection. Thus, there were no insufficient data to perform this meta-analysis. Waldron et al^[30]^ performed a meta-analysis that included 45 studies and found that intravenous glucocorticoids were not accompanied by an increased risk of infection or delayed wound healing. As the included studies pose several procedures and contaminated surgeries, the risk of developing postoperative wound infection cannot be generalized. Toner et al^[31]^ included 56 clinical trials and did not highlight any safety concerns with respect to the use of perioperative glucocorticoids and subsequent infection, hyperglycemia, or other adverse outcomes in elective noncardiac surgery. Other complications, such as gastric ulcers, other site infections (pneumonia, urinary tract infection), and insomnia were not reported in any of the included studies.

Our meta-analysis also has several potential limitations: our analysis comprised only seven RCTs, and the sample size of the included studies was limited; the potential risk of publication bias may exist due to the limited number of included studies; and the follow-up in the included studies ranged from 24 hours to 1 year after THA. Thus, adverse events may have been underestimated. Finally, the different dose and type of glucocorticoids also influence the final conclusion.

## 5 Conclusion

The present meta-analysis demonstrated that intravenous glucocorticoids can alleviate pain, the incidence of PONV, and decrease morphine consumption. Furthermore, the anti-emesis effects were dose-dependent with the dose of glucocorticoids. However, the evidence for its use is limited by the low-quality studies and variation in dosing regimens. Thus, more RCTs are needed to verify the efficacy and safety of intravenous glucocorticoids for THA.
