# Preoperative intravenous glucocorticoids can decrease acute pain and postoperative nausea and vomiting after total hip arthroplasty: A PRISMA-compliant meta-analysis: Erratum

**DOI:** 10.1097/MD.0000000000009728

**Published:** 2018-01-26

In the article, “Preoperative intravenous glucocorticoids can decrease acute pain and postoperative nausea and vomiting after total hip arthroplasty: A PRISMA-compliant meta-analysis”,^[1]^ which appeared in Volume 96, Issue 47 of *Medicine*, the funding information, “The research work was supported by fund Science technology Research Project of Kaifeng City (Grant No:1503115),” was left out.
